# Supplementary material for: Mathematical framework for place coding in the auditory system
Source: PLoS Comput Biol. 2021 Aug 2;17(8):e1009251. doi: 10.1371/journal.pcbi.1009251 (PMC8360601; doi:10.1371/journal.pcbi.1009251)
Supplement: S1 Appendix — Fig. A: Projections of multiple layers of staggered neurons on tonotopic axis decreases Δx. Fig. B: Algebra of loudness summation applied to stimuli consisting of 4 tones with equally spaced frequencies. (PDF) [file pcbi.1009251.s001.pdf]

S1 Appendix

Acoustic Space

The general approach for defining acoustic space is as follows. The elements of acoustic space are pure tones of varying frequencies and sound pressures and may be expressed as a product of frequency space $S^{\mathcal{F}}$ and pressure space $S^{\mathcal{P}}$: $S^{\mathcal{A}} = S^{\mathcal{F}} \times S^{\mathcal{P}}$.

The frequency space ($S^{\mathcal{F}}$) is defined as a half-open interval on the positive real line: $S^F = [f_{min}, f_{max}) \subset \mathbb{R}^+$, where f_{min}, f_{max} are the minimum and maximum audible frequencies, respectively. To account for the fact that discriminability is limited (i.e. tones of similar frequencies are perceived as the same), $S^{\mathcal{F}}$ is partitioned into N_f half-open intervals $h^{\mathcal{F}} = [f_\alpha, f_\beta) = [f_\alpha, f_\alpha + \Delta f)$ where $\Delta f = \frac{1}{N_f}$ is the length of the interval. The use of half-open intervals ensures that there is no 'gap' between adjacent frequency intervals. The set of all half-open intervals is:

$$\mathcal{H}^{\mathcal{F}} = \{h_i^{\mathcal{F}} \mid h_i^{\mathcal{F}} = [f_{min} + i\Delta f, f_{min} + (i+1)\Delta f), f \in S^{\mathcal{F}}, i \in \mathbb{N}_0, i < N_f\} \tag{1}$$

The set of intervals is a partition where each interval is an equivalence class $[f_j]$: for $f_\alpha, f_\beta \in S^F, f_\alpha \sim f_\beta$ if $f_\alpha, f_\beta \in [f_i, f_i + \Delta f)$. This may be extended so that $f_\alpha \sim f_\beta$ if $f_\alpha, f_\beta > f_{max}$ and $f_\alpha \sim f_\beta$ if $f_\alpha, f_\beta \leq f_{min}$. Frequencies f_α, f_β are 'assigned' to f_i if they are between f_i and $f_i + \Delta f$. Thus, f_α, f_β are indistinguishable from each other since both are classified as f_i. Each frequency interval is uniquely identified by the minimum point at the closed end. The set of these points is:

$$\begin{aligned} \mathcal{F} &= \{f_i \mid f_i = f_{min} + i\Delta f, i \in \mathbb{N}_0, i < N_f\} \subset S^{\mathcal{F}} \\ &= \{f_0, f_1, \ldots, f_{N_f - 1}\} \end{aligned} \tag{2}$$

$\mathcal{F}$ is a discretization of $S^{\mathcal{F}}$ and may be thought of as a set of audible frequencies.

The sound pressure space is constructed similarly to yield:

$$a.\quad \mathcal{H}^{\mathcal{P}} = \{h_i^{\mathcal{P}} \mid h_i^{\mathcal{P}} = [p_{min} + i\Delta p, p_{min} + (i+1)\Delta p), p \in S^{\mathcal{P}}, i \in \mathbb{N}_0, i < N_p\} \tag{3}$$

$$b.\quad \mathcal{P} = \{p_0, p_1, \ldots, p_{N_P - 1}\} \subset S^{\mathcal{P}} \text{ (set of physiological pressures)}$$

If only the sets of audible frequencies and pressures are considered, then the discretized acoustic space is:

$$\mathcal{A} = \mathcal{F} \times \mathcal{P} \tag{4}$$

Neural Space

The neural space $S^{\mathcal{X}}$ is constructed in a similar fashion as $\mathcal{F}$ and $\mathcal{P}$. The idea is to partition $S^{\mathcal{X}}$ into subintervals that represent neurons and then to combine the subintervals contiguously to form synaptic intervals. To reflect these properties, the neural space will have a topology that is coarser than the usual topology.

The neural space $S^{\mathcal{X}} \subset \mathbb{R}^+$ is a half-open interval representing the one dimensional tonotopic axis: $S^{\mathcal{X}} = [x_{min}, x_{max})$. $S^{\mathcal{X}}$ is partitioned into N_h half-open intervals, each of width Δx and expressed as $h = [x_\alpha, x_\beta), x_\beta > x_\alpha$ or $[x_\alpha, x_\alpha + \Delta x)$. Each interval represents the length (Δx) of a single cell on the tonotopic axis. The set of intervals is expressed as:

$$\mathcal{H}^{\mathcal{X},1} = \{h_i \mid h_i = [x_{min} + i\Delta x, x_{min} + (i+1)\Delta x), i \in \mathbb{N}_0, i < N_h\} \tag{5}$$

The set $\mathcal{H}^{\mathcal{X},1}$ is a partition, which effectively discretizes $S^{\mathcal{X}}$ (see section above for a similar treatment). Each interval is uniquely identified by the point at the closed end, which also gives it's location along the tonotopic axis. The set $\mathcal{X}$ containing these points is:

$$\begin{aligned} \mathcal{X} &= \{x_i \mid x_i = x_{min} + i\Delta x \mid x_i \in S^{\mathcal{X}}, i \in \mathbb{N}_0, i < N_h\} \\ &= \{x_0, x_1, \ldots, x_{N_h - 1}\} \end{aligned} \tag{6}$$

A synaptic interval is also a half-open interval consisting of a contiguous set of cells. It's length λ is an integral number (n_λ) of cells:

$$\begin{aligned} h_{x,\lambda} &= [x, x+\lambda),\ x \in \mathcal{X}^\lambda,\ 0 < \lambda \leq \lambda_{max} \\ &= [x, x+n_\lambda \Delta x),\ n_\lambda \in \mathbb{N}^+, n_\lambda \leq n_{\lambda,max} \leq N_h \end{aligned} \tag{7}$$

where $n_{\lambda,max}$ is the maximum number of cells allowed in each synaptic interval. To ensure that all synaptic intervals are within $S^{\mathcal{X}}$, the starting points x's are confined to $X^\lambda \subset \mathcal{X}$, which has a 'buffer' that takes into account the maximum synaptic interval length:

$$\mathcal{X}^\lambda = \{x_i \mid i \in \mathbb{N}_0, i \leq (N_h - n_{\lambda,max})\} \subset \mathcal{X} \tag{8}$$

The set containing the possible interval lengths is :

$$\begin{aligned} \Lambda &= \{\lambda_{n_\lambda} \mid \lambda_{n_\lambda} = n_\lambda \Delta x, n_\lambda \in \mathbb{N}^+, n_\lambda \leq n_{\lambda,max} \leq N_h\} \subset S^\Lambda \\ &= \{\lambda_1, \lambda_2, \ldots, \lambda_{max}\} \end{aligned} \tag{9}$$

The set of intervals is expressed as:

$$\mathcal{H}^{\mathcal{X},\Lambda} = \{h_{x,\lambda} \mid h_{x,\lambda} = [x, x+\lambda), x \in \mathcal{X}^\lambda, \lambda \in \Lambda\} \tag{10}$$

A topology $\tau^{\mathcal{X}}$ on $S^{\mathcal{X}}$ is defined as the collection of open sets generated with $\mathcal{H}^{\mathcal{X},1}$ (or $\mathcal{H}^{\mathcal{X},\Lambda}$) as a basis. That is, an element (open set) of $\tau^{\mathcal{X}}$ is any set that is constructed from the union of the Δx intervals. For example, an open set can consist of a single interval made up of a contiguous set of cells (eq. 7) or the union of several such intervals. The topology will be used in developing algebraic operations on intervals (see below).

Subcellular resolution

To facilitate explanation of the model, the Δx was taken to be equal to the width of a single cell. This is true if the divisions of the tonotopic axis are determined by the projections of a single layer of neurons (Fig 2 of main text). Subcellular resolution with a purely place coding scheme may be achieved if the divisions of the tonotopic axis is determined by the projections of multiple layers of neurons (Fig A). If neurons in the different layers are staggered, then there will be more points, with effectively smaller Δx, along the tonotopic axis that can be used to encode frequency. In this scheme, Δx, and hence Δf, can be very small, depending on the neuron density and number of layers. .

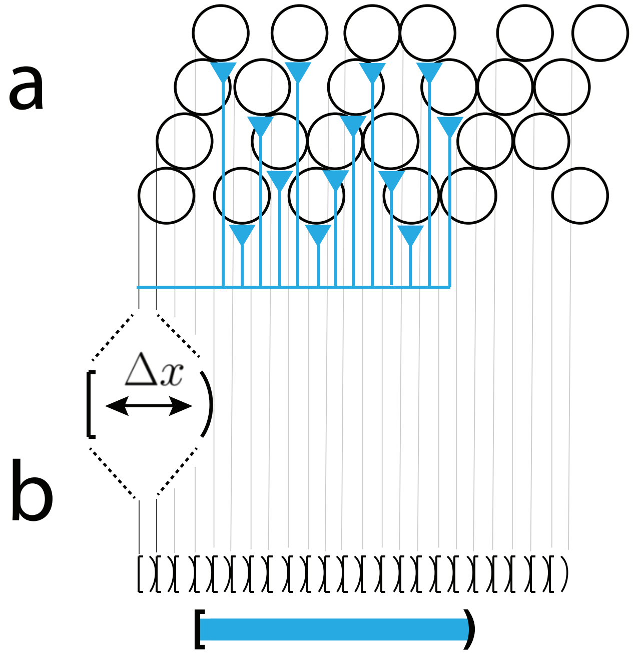

Fig A: Attaining subcellular spatial resolution *a*, multiple layers of staggered neurons. *b*, Projections of individual neurons partition the tonotopic axis, with Δx corresponding to the distance between the nearest neighbors on tonotopic axis. Cyan bar depicts synaptic interval generated by afferents in *a*.

Mapping single elements in acoustic space to neural space

The aim is to construct a mapping between the acoustic and neural spaces in such a way that allows for both encoding and decoding *single* tones in neural space.

Note that a pure tone $a_{f,p}$ corresponds to the ordered pair $(f, p) \in S^{\mathcal{F}} \times S^{\mathcal{P}}$ and an interval

$h_{x,\lambda} = [x,x+\lambda)$ corresponds to the ordered pair $(x,\lambda) \in S^{\mathcal{X}} \times S^{\Lambda}$. The mapping ψ is obtained by first defining mappings from frequency space to neural space and from pressure space to interval length space. After partitioning, the spaces are effectively discretized into $\mathcal{F} \subset S^{\mathcal{F}}, \mathcal{P} \subset S^{\mathcal{P}}, \mathcal{X}^{\lambda} \subset S^{\mathcal{X}}, \Lambda \subset S^{\Lambda}$. These are the functionally and biological relevant sets as they contain the audible frequencies and sound pressures that can be mapped into the compartmentalized neural space. Therefore, the mapping is:

$$\begin{aligned} &\psi_f : \mathcal{F} \to \mathcal{X}^{\lambda}, \psi_f(f) = x; \\ &\psi_p : \mathcal{P} \to \Lambda, \psi_p(p) = \lambda; \\ &\psi_{f,p} : \mathcal{F} \times \mathcal{P} \to \mathcal{X}^{\lambda} \times \Lambda, \\ &\qquad \psi_{f,p}(f,p) = (\psi_f(f), \psi_p(p)) = (x,\lambda) \end{aligned} \tag{11}$$

The maps are bijective if $|\mathcal{F}| = |\mathcal{X}^{\lambda}|$ and $|\mathcal{P}| = |\Lambda|$ and continuous because the discretized acoustic space has the discrete topology.

Algebraic Operations in Neural Space

In this section, the algebraic operations for combining intervals in neural space will be defined. To make the explanations more intuitive, the operations will be developed specifically with open sets in $\tau^{\mathcal{X}}$ with single intervals, though they apply generally to any open set in $\tau^{\mathcal{X}}$.

Addition

Addition ("+") is defined as the union. Given two intervals $h_{x_\alpha \lambda_\alpha}, h_{x_\beta \lambda_\beta} \in \tau^{\mathcal{X}}$:

$$\begin{aligned} &+ : \tau^{\mathcal{X}} \times \tau^{\mathcal{X}} \to \tau^{\mathcal{X}} \\ &h_{x_\alpha \lambda_\alpha} + h_{x_\beta \lambda_\beta} \stackrel{\text{def}}{=} h_{x_\alpha \lambda_\alpha} \cup h_{x_\beta \lambda_\beta} \end{aligned} \tag{12}$$

The addition operation yields a single interval if the intervals overlap and the same two intervals if the intervals are disjoint.

$$h_{x_\alpha\lambda_\alpha} + h_{x_\beta\lambda_\beta} = \begin{cases} h_{x\lambda}, x = \min(x_\alpha, x_\beta), \lambda = |h_{x\lambda_\alpha}| + |h_{x\lambda_\beta}| - |h_{x\lambda_\alpha} \cap h_{x\lambda_\beta}|, & h_{x\lambda_\alpha} \cap h_{x\lambda_\beta} \neq \emptyset \\ \{h_{x_\alpha\lambda_\alpha}, h_{x_\beta\lambda_\beta}\} & h_{x\lambda_\alpha} \cap h_{x\lambda_\beta} = \emptyset \end{cases} \tag{13}$$

For more than two intervals, the total length λ is calculated using the Inclusion-Exclusion principle.

The algebraic structure with the addition operation is a commutative monoid since it inherits the algebraic properties of the union operation. In particular, addition is closed because the result is either a longer, half-open interval or two disjoint half-open intervals, either of which is an element of $\tau^{\mathcal{X}}$. Addition is commutative, associative, has the empty set as the identity element, and does not have an inverse.

Multiplication

Multiplication "·" of synaptic intervals is defined as the set minus operation ($\setminus$). Given two intervals $h_{x_\alpha\lambda_\alpha}, h_{x_\beta\lambda_\beta} \in \tau^{\mathcal{X}}$, where $h_{x_\alpha\lambda_\alpha}$ is the multiplier and $h_{x_\beta\lambda_\beta}$ is the multiplicand,

$$\begin{aligned} &\bullet : \tau^{\mathcal{X}} \times \tau^{\mathcal{X}} \to \tau^{\mathcal{X}}; \\ &h_{x_\alpha\lambda_\alpha} \cdot h_{x_\beta\lambda_\beta} \overset{\text{def}}{=} h_{x_\beta\lambda_\beta} \setminus h_{x_\alpha\lambda_\alpha} \end{aligned} \tag{14}$$

Multiplication can yield several results.

$$h_{x_\alpha,\lambda_\alpha} \cdot h_{x_\beta,\lambda_\beta} = \begin{cases} 1.\, h_{x_\beta,\lambda_\beta}, & h_{x_\alpha,\lambda_\alpha} \cap h_{x_\beta,\lambda_\beta} = \emptyset \\ 2.\, [x_\beta, x_\alpha), & h_{x_\alpha,\lambda_\alpha} \cap h_{x_\beta,\lambda_\beta} \neq \emptyset, x_\beta < x_\alpha, h_{x_\alpha,\lambda_\alpha} \not\subset h_{x_\beta,\lambda_\beta} \\ 3.\, [x_\delta, x_\delta + |h_{x_\beta,\lambda_\beta} \setminus h_{x_\alpha,\lambda_\alpha}|), & h_{x_\alpha,\lambda_\alpha} \cap h_{x_\beta,\lambda_\beta} \neq \emptyset, x_\beta \geq x_\alpha, h_{x_\beta,\lambda_\beta} \not\subset h_{x_\alpha,\lambda_\alpha} \\ \quad \text{with } x_\delta = x_\beta + (\lambda_\beta - |h_{x_\beta,\lambda_\beta} \setminus h_{x_\alpha,\lambda_\alpha}|) & \\ 4.\, \emptyset, & h_{x_\beta,\lambda_\beta} \subset h_{x_\alpha,\lambda_\alpha} \\ 5.\, \{[x_\beta, x_\alpha), [x_\alpha + \lambda_\alpha, x_\beta + \lambda_\beta)\}, & h_{x_\alpha,\lambda_\alpha} \subset h_{x_\beta,\lambda_\beta} \end{cases} \tag{15}$$

The algebraic structure with the multiplication operation is a magma, with properties inherited from the set minus operation. Multiplication is closed because the set minus operation yields an interval that is either the empty set or a half-open interval(s) with a minimum length(s) of Δx and maximum length of λ_{max}. In either case, the resulting interval is in $\tau^{\mathcal{X}}$. While the $\emptyset$ is a left identity element, there is no unique inverse element since the result is the $\emptyset$ whenever the length of multiplier is greater than or equal to that of the multiplicand. It is also neither commutative nor associative.

Proposition 0.0.1. The *multiplication* operation is left but not right distributive over addition.

Proof. **Left distributive**: Let $h_{x_\alpha,\lambda_\alpha}, h_{x_\beta,\lambda_\beta}, h_{x_\gamma,\lambda_\gamma} \in \tau^{\mathcal{X}}$

$$\begin{aligned} &= h_{x_\gamma,\lambda_\gamma} \cdot (h_{x_\alpha,\lambda_\alpha} + h_{x_\beta,\lambda_\beta}) \\ &= (h_{x_\alpha,\lambda_\alpha} \cup h_{x_\beta,\lambda_\beta}) \setminus h_{x_\gamma,\lambda_\gamma} \\ &= h^c_{x_\gamma,\lambda_\gamma} \cap (h_{x_\alpha,\lambda_\alpha} \cup h_{x_\beta,\lambda_\beta}),\ \text{'c' is complement} \\ &= (h^c_{x_\gamma,\lambda_\gamma} \cap h_{x_\alpha,\lambda_\alpha}) \cup (h^c_{x_\gamma,\lambda_\gamma} \cap h_{x_\beta,\lambda_\beta}) \\ &= h_{x_\alpha,\lambda_\alpha} \setminus h_{x_\gamma,\lambda_\gamma} \cup h_{x_\beta,\lambda_\beta} \setminus h_{x_\gamma,\lambda_\gamma} \\ &= h_{x_\gamma,\lambda_\gamma} \cdot h_{x_\alpha,\lambda_\alpha} + h_{x_\gamma,\lambda_\gamma} \cdot h_{x_\beta,\lambda_\beta} \end{aligned}$$

Not Right distributive:

$$
\begin{aligned}
&(h_{x_\alpha,\lambda_\alpha} + h_{x_\beta,\lambda_\beta}) \cdot h_{x_\gamma,\lambda_\gamma} \\
&= h_{x_\gamma,\lambda_\gamma} \setminus (h_{x_\alpha,\lambda_\alpha} \cup h_{x_\beta,\lambda_\beta}) \\
&= (h_{x_\gamma,\lambda_\gamma} \setminus h_{x_\alpha,\lambda_\alpha}) \cap (h_{x_\gamma,\lambda_\gamma} \setminus h_{x_\beta,\lambda_\beta}) \\
&\neq h_{x_\alpha,\lambda_\alpha} \cdot h_{x_\gamma,\lambda_\gamma} + h_{x_\beta,\lambda_\beta} \cdot h_{x_\gamma,\lambda_\gamma}
\end{aligned}
$$

□

Comparison with columnar organization

In the mathematical model, the 'functional' unit is an interval that can vary in size, depending on the sound intensity. The indistinct borders are consistent with topographical organization of afferents [1]. The expanding border facilitates high resolution representation of both frequency and pressure. Assuming a bijective mapping between acoustic and neural spaces, the number of pressure levels that can be represented is $|\mathcal{P}| = |\Lambda| = n_{\lambda,max}$ and the number of frequency levels is given by: $|\mathcal{F}| = |\mathcal{X}^{\lambda}| = N_h - n_{\lambda,max} + 1$. The relation between the number of frequency and pressure levels is then : $|\mathcal{F}| = N_h - |\mathcal{P}| + 1$. The total number of neurons needed to achieve an equal number of frequency and pressure levels ($|\mathcal{F}| = |\mathcal{P}| = n_{levels}$), is $N_h = |\mathcal{F}| + |\mathcal{P}| - 1 = 2 \times n_{levels} - 1$.

In contrast, consider an alternative neural space that consists of classical columns [2]. The definition of a column has evolved [3] but for illustrative purposes, columns are defined here as having distinct borders; the enclosed neurons have identical receptive fields. In this scheme, the location of the active column encodes frequency and sound pressure is encoded as the number of neurons within each column that become active during the stimulus (assuming a brief stimulus so that neurons are effectively binary). For example, the probability of neurons firing within a column may increase with sound pressure (e.g. population rate code).

The neural space consists of a set of adjacent columns, each of which is a half-open interval $[x, x + n_w \Delta x)$ where n_w is the number of cells along the tonotopic axis. The number of frequency levels that can be represented is equal to the number of columns that fit along the tonotopic axis: $|\mathcal{F}| = \frac{N_h}{n_w}$. The number of pressure levels that can be represented is equal to the number

of cells in each column: $|\mathcal{P}| = n_w$. Hence, the relationship between the number of frequency and pressure levels is: $|\mathcal{P}| = \frac{N_{total}}{|\mathcal{F}|}$ where N_{total} is the total number of neurons and is equal to N_h for a monolayer column and equal to $N_h \times n_z$ if each column is 2 dimensional and has a height of n_z cells. The total number of neurons needed to achieve an equal number of frequency and pressure levels ($|\mathcal{F}| = |\mathcal{P}| = n_{levels}$), is $N_h = n_{levels}^2$ (or $N_h \times n_z = n_{levels}^2$ for a column with height n_z).

Algebra of loudness summation

A multi-frequency stimulus is a set of tones with increasing frequencies $F_m = \{f_i \mid i \in I\}$ where $I = \{j, \ldots, n), j \geq 0, n \leq N_f$. In neural space, the stimulus generates a set of excitatory intervals H_m that is the union of the individual intervals generated by each tonal component: $H_m = \bigcup_{i \in I}(h_i^E = [x_i, x_i + \lambda))$, where λ is the length of each interval.

Definition 0.0.1. An inhibitory (half open) interval h^I is in the lateral inhibitory configuration with an excitatory (half open) interval h^E, if $h^I \cap h^E = \emptyset$ and there exists a half-open interval $\hat{h} \supset (h^E \cup h^I)$ so that if $x \in \hat{h}$, then $x \in h^E \cup h^I$. This ensures that the excitatory and inhibitory intervals abut each other with no gap. Note that this definition applies whether h^I is to one side of h^E or is split into two that occupies either side (as in the main text and Proposition 0.0.2 above) $h^I = h_L^I \cup h_R^I$.

Definition 0.0.2. A tone in multi-frequency stimulus is 'dominant' if it results in an excitatory interval h_d^E and an inhibitory interval h^I that is much larger than that generated by the other tones.

Physiologically, the dominant frequency f_d could be the center frequency that identifies a critical band (CB) [4] or the lowest frequency tone of a complex stimulus [5]. It is assumed that every multi-frequency stimulus has a dominant frequency, which generates the synaptic interval $h_d^E \subset H_m$. The non-dominant frequencies may also generate associated inhibitory intervals but in the following analyses, they are assumed to be much weaker than that generated by the dominant frequency and can be ignored.

The net excitatory interval in the presence of inhibition is given by the algebraic operation: $h_l = h^I \cdot H_m$. The length $|h_l|$ is taken to be the proxy for loudness perception.

Proposition 0.0.2. In a lateral inhibition configuration consisting of the dominant excitatory interval h_d^E and an inhibitory interval h^I, $|h_l|$ does not change when the size (number of element and/or length of each element) of H_m is increased as long as H_m remains within (is a subset of) a 'critical interval' $H_{CI} = h_d^E + h^I \stackrel{\text{def}}{=} h_d^E \cup h^I$ and increases thereafter.

Proof. First note that $H_m = h_d^E \cup (H_m \setminus h_d^E) = h_d^E + h_d^E \cdot H_m$ since $h_d^E \in H_m$. Thus,

$$h_l = h^I \cdot H_m = h^I \cdot (h_d^E + h_d^E \cdot H_m) = h^I \cdot h_d^E + h^I \cdot (h_d^E \cdot H_m) \tag{16}$$

$$= h_d^E + h^I \cdot (h_d^E \cdot H_m) \text{ since } h_d^E \cap h^I = \emptyset$$

$$\text{Therefore, } |h_l| =$$

$$\begin{cases} |h_d^E|, & h_d^E \cdot H_m \subset h^I \\ > |h_d^E|, & h_d^E \cdot H_m \not\subset h^I \end{cases}$$

$$\text{Now, } h_d^E \cdot H_m \subset h^I \Rightarrow h_d^E + (h_d^E \cdot H_m) \subset h_d^E + h^I \text{ or } H_m \subset H_{CI}$$

Thus, h_l is equal to h_d^E as long as $H_m \subset H_{CI}$ and increases when $H_m \not\subset H_{CI}$. □

Definition 0.0.3. A set of tones with frequencies given by F_m, each delivered at some sound pressure level, is represented in neural space by the set of intervals given by H_m. The critical band (CB) is difference between the highest frequency and lowest frequency in F_m that satisfies $H_m \subseteq H_{CI}$.

An increase in stimulus intensity (or sound pressure) results in a lengthening of excitatory intervals. Hence, the lengths of h_d^E and the other excitatory intervals of H_m will increase.

Proposition 0.0.3. The critical band does not change with stimulus intensity provided that the lateral inhibitory configuration is preserved and the length λ_I of the inhibitory interval h^I remains constant.

Proof. We need to show that $H_m \subset H_{CI}$ when the length of each interval of H_m is increased by an amount Δh due to the increase in sound pressure level of each tonal components of the stimulus. Recall that a set of tones with frequencies in F_m results in a set of intervals H_m where one of the intervals h_d^E is dominant.

The critical interval H_{CI}, as defined above, consists of the dominant excitatory interval and an inhibitory interval in the lateral inhibitory configuration: $H_{CI} = h_d^E \cup h^I$. If $H_m \subset H_{CI}$, then $|H_{CI}|$ must be greater than or equal to the distance $\delta = (x_n + \lambda) - x_1$, where x_1, x_n are, respectively, the leftmost points of the first and last interval in H_m: $|H_{CI}| = |h_d^E| + |h^I| = \lambda + \lambda_I \geq \delta$.

Increasing the length of each interval (which includes h_d^E) by Δh extends the last point in H_m so that $\delta = (x_n + \lambda + \Delta h) - x_1$. Under the condition that the lateral inhibitory configuration is preserved (h_d^E and h^I remain disjoint) and the length of the inhibitory interval is constant, the length of H_{CI} increases by the same amount: $|H_{CI}| = (\lambda + \Delta h) + \lambda_I$. Therefore, $H_m \subset H_{CI}$ □

The total length (h_l) resulting from the operations are shown graphically for band-limited white noise with increasing bandwidth (main text) and for a complex stimulus with 4 tones with increasing spacing between frequencies (Fig B)

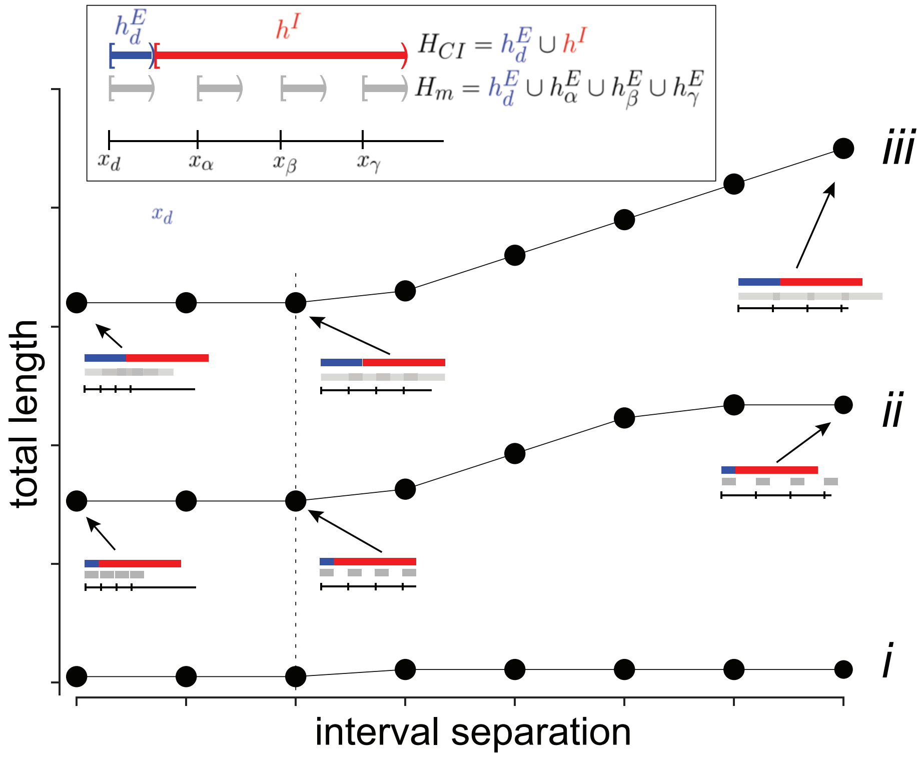

Fig B: Algebra of loudness summation Predicted interval lengths resulting from the interaction of 4 tones delivered simultaneously (inset). *Boxed inset*, The tones generate 4 excitatory intervals (H_m, gray) one of which is dominant (h_d^E, *blue*). The dominant tone also generates an inhibitory synaptic interval (red). Plot shows resultant length (h_l) after the operations as the frequency spacing between intervals in H_m is increased (abscissa). Dotted vertical line marks deviation from constant value. Compare with Fig 3 of [6]

References

1. Towe AL. Notes on the Hypothesis of Columnar Organization in Somatosensory Cerebral Cortex; pp. 32–47. Brain, Behavior and Evolution. 1975;11(1):32–47.

2. Mountcastle VB. Modality and topographic properties of single neurons of cat's somatic sensory cortex. Journal of neurophysiology. 1957;20(4):408–434.

3. The cortical column: a structure without a function. Philosophical Transactions of the Royal Society B: Biological Sciences. 2005;360(1456): 837-862.

4. Fastl H, Zwicker E. Psychoacoustics: facts and models. vol. 22. Springer Science & Business Media; 2006.

5. Wegel R, Lane C. The auditory masking of one pure tone by another and its probable relation to the dynamics of the inner ear. Physical review. 1924;23(2):266.

6. Zwicker E, Flottorp G, Stevens SS. Critical band width in loudness summation. The Journal of the Acoustical Society of America. 1957;29(5):548–557.
