# Supplementary material for: Mathematical framework for place coding in the auditory system
Source: PLoS Comput Biol. 2021 Aug 2;17(8):e1009251. doi: 10.1371/journal.pcbi.1009251 (PMC8360601; doi:10.1371/journal.pcbi.1009251)
Supplement: Attachment — Submitted filename: Response_to_Reveiwers.pdf [file pcbi.1009251.s002.pdf]

I thank the referees for their careful reading of the manuscript and helpful critiques that will improve the manuscript. I've addressed the referee's major concerns and critiques, which were mainly about the Discussion section and supplementary material.

Response to Referee 1's concerns

I thank the referee for carefully reading the manuscript and making very focused comments that helped clarify confusing elements of the manuscript.

1. The work aims to illustrate the conditions that support a place code for frequency and intensity. However, the results do not show the code performing in a way that matches or predicts psychophysical measurements. In describing equation 4 (line 120) it is stated that the mapping can be made to be bijective by adjusting the number of elements in each space. This can certainly be done if there are no constraints.

The goal was to construct a model starting with fundamental mathematical principles with minimal reliance on data, which are often disputed or incomplete. Therefore, the constraints are set by rules of e.g. set theory, topology and algebra. Bijective mappings were chosen because they seem ideal both for encoding and decoding signals. Having defined synaptic intervals as the functional units, it was determined that the range of frequencies and sound pressures needed to preserve bijective maps were set by the number of cells along the tonotopic axis and the maximum number of cells in a given synaptic interval (line 126 of revised manuscript). I am not aware of any experiment that has measured the relation between these parameters and the range of audible frequencies and sound pressures.

2. Is it possible to start from psychophysical measurements and predict something about the neural representation, or vice versa?

In the revised manuscript, I added a new section (**Algebra of loudness summation**) (starting on line 414) where I applied the analyses to loudness summation. I developed a formula with the algebraic operations that describes a key feature of loudness summation: increasing the bandwidth of a white noise stimulus does not increase the perceived loudness until the bandwidth exceeds a 'critical band' (see Figure below). The analyses suggest network configurations that could underlie the critical band and could account for the fact that it doesn't change with stimulus intensity. The strength of the analyses is that the terms used and conditions where the formula are valid can be defined precisely with proofs (lines 115-156 of supplementary text S1). I also show that the formula generalizes to a different experiment where multiple tones are delivered instead of white noise (Supplementary Figure S2).

Also, in the revised manuscript, there is now a section entitled **"Place code framework in auditory processing: evidence and implications"** (starting in line 319). In this section, I discuss perceptual data in the context of the model. For details, please see point 3 of my responses to Referee's 3 concerns below.

3. A fundamental aspect of the analysis is the partitioning of the frequency and pressure spaces into a discrete set of equivalence classes. However, I have trouble understanding the definition of the equivalence classes in relation to the notion of perceptual discriminability. For example, in the equivalence classes f_{min} and $f_{min} + df - epsilon$ are equivalent for any $0 < epsilon <= df$, but $f_{min} + df - epsilon$ and $f_{min} + df$ are different for any $epsilon > 0$. This says that the JND is essentially 0 for frequencies at the boundary of the intervals. This would also suggest that

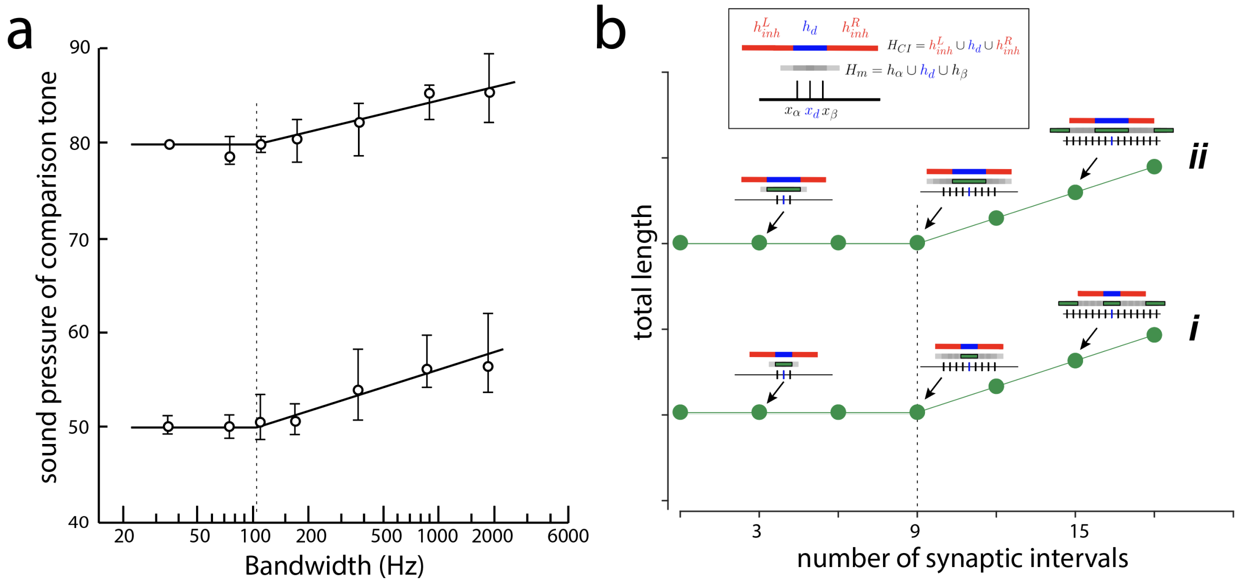

Algebra of loudness summation ***a***, Perceived loudness when subjects were presented with white noise of increasing band width (abscissa) at 2 sound pressure levels (lower and upper traces). ***b***, Predicted interval lengths resulting from the interaction of multi-tone stimulus delivered simultaneously. ***Boxed inset***, overlapping synaptic intervals (H_m, gray) generated by stimulus with 3 frequency components. The dominant tone (blue) also generates two inhibitory side bands (red). Plot shows resultant length (h_l) after the operations (see text) as the number of intervals in H_m (or number of frequency components) is increased (abscissa).

JND measurements should show a strong asymmetry for test frequencies above or below a reference frequency. Please clarify how the defined equivalence classes relate to perceptual discriminability.

This is a very good point. To recapitulate, the JND should depend on how near the frequencies (say 2 frequencies f_α, f_β are to closed end of the interval (say f_{min}) that defines the equivalence class ($[f_{min}, f_{min} + \Delta f)$. If f_α, f_β are near f_{min}, then the empirically-measured JND should be relatively large since f_β would have to increase by nearly Δf before it can be distinguished from f_α. In contrast, JND should be small if f_α, f_β are near $f_{min} + \Delta f$. If this were the case, then the JND should theoretically range from being infinitesimally small to being equal to Δf since a small change in f_β puts it in the next equivalence class. I am not aware of any study that tests this prediction systematically. The appropriate experiment would be to ask subjects if they can discriminate two tones with frequencies (f_α, f_β) that differ by an ϵ and then to increase both frequencies in small increments (keeping ϵ fixed). The prediction is that the subject cannot discriminate between f_α and f_β except at regular intervals of Δf.

In the revised manuscript, the relation between Δf and frequency difference limens are discussed (lines 352-363). In the Results (line 115), I now refer to Δf as being 'loosely' related to the limen and direct the reader to the Discussion for the explanation.

4. The nature of noise in the model is unclear. The input to the network is described as Gaussian distributed. Are the inputs drawn from a Gaussian distribution, or does the strength of the input follow the shape of a Gaussian curve? Please discuss the nature of noise in the model and how the model may be limited if noise is not considered.

The average number ($n_{inputs}(x)$) of inputs that each neuron at location x receives follows a Gaussian curve so that cells at the center of the network received more inputs. For each run, the number was randomized by drawing a number from a Gaussian distribution with mean equal to the $n_{inputs}(x)$ and a standard deviation of $0.25 * n_{inputs}(x)$. The stimuli were brief (cells fired a single action potential) so the effects of the noise did not accumulate significantly with time but did introduced trial-to-trial variability in the number of activated neurons, which is more in vivo-like. These points were clarified in the Methods section (lines 547-551 of the revised manuscript).

Without the added variability, there will be a sharp border across the tonotopic axis separating neurons that fired from those that did not. The trends plotted in Figs. 6-8 are likely to persist though the curves may be less smooth.

5. The simulation with spiking neurons tests the predictions of the addition and multiplication operations on an adaptive exponential integrate and fire model with parameters that are based on pyramidal neuron responses. Given the diversity of response types in the auditory system, the simulation does not appear to be a sufficient test of the model as a general place coding model that would apply throughout the auditory system. Is it intended that the model applies specifically to a population of neurons with the parameters used in the simulation? It would strengthen the results to show that the spiking simulation is robust to changes in neuron parameters.

The simulations were purposely run with short (50 ms) duration stimulus so that neurons fired only a single action potential. As stated in the introduction (line 38), many auditory processes require only brief stimulus (as short as 5 ms). The analyses treat neurons as binary units so only the first spike is important. Most of the differences in cellular properties (e.g. activation/inactivation of voltage gated channels) that affect firing are manifested after the first spike. Hence, the response of a repetitively firing cell in cortex and that of a bushy cell in brainstem that fires only a single action potential will be qualitatively similar for brief stimuli provided the inputs are suprathreshold. Therefore, the results should generalize across cell types although there may be quantitative differences owing to differences thresholds and passive properties. Longer duration stimuli will accentuate differences between cells, as well as recruit recurrent activity in the network. The math will need to be adjusted later to incorporate network dynamics. The generality of the model is now discussed (lines 526-533) of the revised manuscript).

6: Line 92: Please discuss why dx is constant. Shouldnt dx depend on x if different frequency ranges are differentially represented in the neural space?

The Δx of neural space represent the space taken up by a single neuron (line 94 of revised manuscript) and so should be constant throughout the tonotopic axis. To simplify analyses, the model ignores the increase in frequency and pressure ranges. However, this can be easily implemented by transforming frequency space to octaves and sound pressure to have units of decibels prior to mapping to neural space. The analyses were intended to be abstract and so specific details were purposely left out so as to highlight the underlying mathematics. That an octave or decibel scale is more appropriate to capture the true range of hearing is now discussed (lines 504-507 of the revised manuscript).

7: Lines 107-109: It is stated that frequency/pressure takes on an uncountable number of values, but the audible range is bounded. A bounded interval is still uncountable. Did you mean unbounded instead of uncountable, or is the discretization of frequency/pressure what is being referenced here?

Thank you for pointing this out. The text in the revised manuscript (line 109) was changed to "Theoretically, frequencies and pressures are unbounded and can take an uncountable number

of values but under physiological conditions, the audible range is likely bounded by minimum and maximum values and is finite"

8: Line 113: Please discuss the motivation for df and dp being independent of f and p, respectively, when the just noticeable difference frequency and pressure depend on the reference frequency and pressure.

Δf and Δp are no longer considered to be directly related to the JNDs for the reasons stated in the comments of point 3 above. Aside from that, Δf and Δp were kept constant to simplify the analyses. It would have been cumbersome to incorporate varying values for both and would be an added complication to an already difficult manuscript. Having established the mathematical framework, it should be relatively simple to incorporate non-constant Δf and Δp in future studies.

9: Lines 248-251: It is stated that the prediction was for no change when σ_α was smaller than σ_α, but in parentheses this is written as ($\sigma_\alpha/\sigma_\beta < 0.5$). Should the condition ($\sigma_\alpha/\sigma_\beta < x$) be equivalent to the condition that is written?

The section of the sentence that reads "was smaller than σ_β ($\frac{\sigma_\alpha}{\sigma_\beta} < 0.5$)" was removed (line 258 of revised manuscript).

10: Supplementary material equation 1, equation 4a: It appears that the intervals hf and hp have f and p as arguments, respectively. The author might consider different notation for the frequency and pressure intervals because it is not clear whether h2 is a frequency or pressure interval, for example. There is a similar ambiguity for Hf and Hp. It is typically clear from the context what is being referred to, but the author might consider revising the notation. Superscript F and P in h's

Thank you for the suggestion. I changed the notation so that the space is in superscripts. For example, $\mathcal{H}_\mathcal{F}$ was changed to $\mathcal{H}^\mathcal{F}$

11: Supplementary material equation 18 bottom: I believe that af should be afalpha or afbeta.

The condition was that $f_\alpha = f_\beta = f$ so f is a generic variable. However, in response to referee 2, I removed the equation and section from the supplementary material.

Response to Referee 2's concerns

I thank the referee for his/her critical comments, which indicated that I failed to convey the most important features of the model, particularly in the Discussion. A major concern of the referee was equation 18 in the supplementary material, which raised doubts in his/her mind as to the validity of the entire mathematical model. As I point out below, the equation is not essential to the manuscript and to avoid controversy, I deleted it in the revised version of the manuscript. The other critique was that the significance of the model was not clearly stated, in particular, I incorrectly described the assumptions of the model as findings. I re-organized and re-wrote the discussion to emphasize the significance and implications of the model.

1.This alone [eq. 18] seems to fatally undermine the rest of the paper. If something as basic as adding two tones together isn't handled correctly....(2nd half addressed below)..

The original purpose of equation 18 was to develop a homomorphism between acoustic and neural spaces, which required defining algebraic operations. Ultimately, I decided not to pursue the topic but inadvertently left the equation and section in the supplementary material. It was not referenced in the main text and, regardless of whether it is correct or not, is inconsequential to the analyses. I therefore deleted it in the revised manuscript. Doing so has no effect on the conclusions of study because the algebraic operations described in the main text take place in neural space and it makes no difference to the analyses how tones were combined in acoustic space. Put another way, the cochlea sees only a tone ($tone_{f,p}$) with frequency f and amplitude p even if it was assembled from two tones with the same frequency but different amplitudes and phases ($tone_{f,p} = tone_{f,p_\alpha,\phi_\alpha} + tone_{f,p_\beta,\phi_\beta}$). For the referee's benefit and to respond to his/her specific comments, I give below the rationale for equation 18 and why it was appropriate for the intended abstract application .

2. Firstly, there appear to be some fundamental problems in terms of the definition of the acoustic space. As far as I understand, the acoustic space is defined as a discrete 2d grid where a given pure tone of frequency f and amplitude p is mapped to a point in this grid. A pure tone is then represented essentially by a matrix where all elements are 0 except a single element which is 1 (I state it like this because it seems much simpler to get your head round that than the version in the paper, and entirely equivalent).

If the referee agrees that they are equivalent, I would prefer to keep the more formal notation used in the manuscript. I don't go into the formal definition in the main text and instead use a graph (Fig. 2D) as a visual aid. The use of matrices with zeros and ones is also somewhat cumbersome because the indices for the rows and columns are natural numbers rather than reals. Also, matrices tend to be associated with vector spaces and could create some confusion as many of the linear algebraic operations are not valid in the place coding scheme.

3. If we were only talking about a single pure tone, then the stimulus space would be the set of all matrices with this property. However, it seems that combinations of tones are allowed, and an addition operation is defined on the acoustic space in the supplementary material (eqs 17-18), so that simultaneously playing two tones at different frequencies and amplitudes gives you a matrix with two 1s. However, there is a major problem here that can be illustrated with the very simple example of adding two tones at the same frequency. Two tones with the same frequency f and amplitude p added together will give a new tone with the same frequency f and depending on their phase relationship any amplitude between 0 (out of phase) to 2p (in phase). Equation 18 only allows for an output amplitude of p. I am not sure therefore what the addition operation in the acoustic space is supposed to represent, but it isn't sounds played simultaneously.

Equation 18 describes an abstract way of combining tones with different frequencies in acoustic space. The referee's comment relates to the special case where two tones have the same frequency. In this instance, the standard definition of summation takes into account the phase difference between the tones. For purposes of the abstract mathematical analyses, I opted not to use this for 2 reasons.

a. In a purely place coding scheme, there is no variable that describes time; more specifically, there is no term for phase. Eventually, the model can be extended to include time but it is beyond the scope of the present study.

b. In defining algebraic operations, it is important to satisfy the closure property (operations on a set produces a result within the set). To represent audible sound pressures, the pressure space is bounded by minimum (p_{min}) and maximum (p_{max}) values. Therefore, if the two sinusoids have the same frequency, no phase difference, and amplitudes equal to p_{max}, the result is a sinusoid whose amplitude is greater than p_{max}. To satisfy closure, I instead defined the addition of two tones with the same frequencies as being equal to the tone with the larger amplitude (eq. 18). Since the amplitude of each sinusoid is within p_{min} and p_{max}, the sum as defined is always within the set.

4. The discussion (L311-319) states that three key findings, but none of them are findings, they are rather the definitions of the model. There is no 'finding' that neural space is a set of contiguous neurons, it's the definition of the model and it is not an accurate model. There is no 'finding' that operations in neural space must be union and set difference, it's another definition of the model.

I thank the referee for pointing this out (referee 3 made the same comment). The aim of the study was to introduce a formalism for place coding. There are no 'findings' in the sense that there wasn't a specific hypothesis that was being tested. The analyses do have significant implications for auditory processing and there are experimental evidence (electrophysiological and psychophysical) that the algebraic operations are occurring during auditory processing (summarized in my response to point 5 below). I changed "key findings" to " key feature" (line 325 of revised manuscript).

5. .... we would need a very strong argument and clear reasoning that this framework has anything to say about real auditory perception, and there is no such argument in the paper

I re-wrote and re-organized the Discussion to highlight what the model says about auditory perception, which were scattered and likely difficult to find in the original manuscript. In the revised version, there is now a section entitled **"Place code framework in auditory processing: evidence and implications"** (starting in line 319) that discusses the significance and implications of the analyses. To summarize the content of this section:

a. Functional units with flexible borders allows for high resolution representation of *both* frequency and sound pressure. To show this, I compare the theoretically attainable number of frequency and pressure levels with functional units that resemble classical cortical columns (lines 332-351).

b. The algebraic operations are consistent with experimental data and in some cases offer alternative interpretations. Consistent with the addition operation (defined as the union) are observations that two tones with similar frequencies are perceived as a single tone (line 369) and binaural inputs (electrophysiological and psychophysical) sum sublinearly (lines 371-380). The sublinear summation does not necessarily require inhibition. The effect of the multiplication operation (defined as the set minus) is consistent with the increase in receptive fields of neurons after

inhibition is blocked (line 384). The manner in which multiplication distributes over addition suggests ways of designing experiments to examine the effects of multiple excitatory and inhibitory inputs (lines 388-405). Finally, the discretization of frequency space (via equivalence classes) is related to experimentally-measured just noticeable difference in frequency (or frequency difference limens).

c. The formal description of the neural space that supports a place code can be used to construct mappings to other brain regions along the auditory pathway (lines 406-413). It potentially sets constraints on the anatomical organization of neural spaces that will support a desired mapping.

d. The formalism is used to describe a perceptual phenomena and to infer possible network mechanisms. Algebraic operations are derived that describe loudness summation (lines 414-475), leading to a possible circuit interpretation of the so-called "critical band". Proofs in the supplementary material (lines 115-156) defines precisely the terms and sets the conditions where the formula applies. See my response to comment 5 for details.

5. I'm generally very sympathetic to what I see as the aim of this paper, which is to make a mathematical model which - even though it oversimplifies - is able to throw light on some fundamental aspect of the problem. However, I don't see any of that here. .

In the revised manuscript, I added a new section (**Algebra of loudness summation**) (starting on line 414) where I applied the analyses to throw light on a well-documented perceptual phenomenon. I developed a formula with the algebraic operations that describes a key feature of loudness summation: increasing the bandwidth of a white noise stimulus does not increase the perceived loudness until the bandwidth exceeds a 'critical band' (see Figure below). The analyses suggest network configurations that could underlie the critical band and could account for the fact that it doesn't change with stimulus intensity. The strength of the analyses is that the terms used and conditions where the formula are valid can be defined precisely with proofs (lines 115-156 of supplementary text S1). I also show that the formula generalizes to a different experiment where multiple tones are delivered instead of white noise (Supplementary Figure S2).

Response to Referee 3's concerns

The referee's primary criticism was that the significance of the model was not made clear in the manuscript. The stated findings were actually just the assumptions and definition of the model. Moreover, some of the conclusions seemed trivial. I thank the referee for these very helpful and insightful criticisms.

1.It is somewhat unclear what the main findings are, and there is substantial confusion between what the author calls findings of the study and what are actually just the definitions and assumptions of the model that the author proposes. Key finding are outlined in lines 311-319 of the discussion. There are three. The first finding about the function unit with flexible borders (lines 311-314) is not truly a finding, but a definition of the mathematical framework proposed by the author.

I thank the referee for pointing this out (referee 2 had a similar comment). I agree completely that what I list as 'findings' are actually features and assumptions of the model. I changed "key findings" to " key feature" (line 325 of revised manuscript). Importantly, I re-wrote and re-organized the Discussion to highlight the significance and applicability of the formalism to auditory processing (detailed in my response to comment 3 below).

2. The second finding about the range and resolution dictated by the network architecture (lines 314-316) is a trivial consequence of using a place code: It is a given that network architecture in which different neurons respond to different frequencies will dictate the limitations of the code. In fact, the author outlines the intuition behind this conclusion in the Introduction.

The sentence was poorly constructed. The range and resolution are dictated by the *mathematical* and not by the *network* architecture. The limitation is imposed by the topology of the neural space as it determines the number of frequencies and sound pressure levels that can be mapped bijectively. For comparison, I calculated the frequency and sound pressure resolutions that can be obtained with a columnar organization (lines 335-345 of old manuscript, lines 332-351 in revised version, lines 88-113 of supplementary text S1). For a columnar organization (combined with population rate code to represent sound pressure), the number of frequency levels that can be represented is inversely related to the number of pressure levels (line 344). Ideally, the size of the column should approach 1 cell but this would be at the expense of the number of pressure levels (see my response to point 6 for further explanation). With flexible borders, both frequency and intensity can be represented at relatively higher resolution than with columns.

3. It is not clear whether these operations are simply a formalism to describe the abilities and limitations of the place code, or whether these algebraic operations provide a new insight into these abilities and limitations.....Perhaps the manuscript is not intended to outline findings about neural networks, but is rather intended to introduce a formalism that may be useful for describing place coding. In this case, it is unclear in what way this formalism is useful. Does it allow describing phenomena that could not be described before? Does it reveal insights or make predictions about auditory circuits?

As suggested out by the referee, the study is indeed not intended to test any specific hypothesis about neural networks but instead to introduce a formalism. It is clear that the Discussion of the original manuscript failed to show what the formalism is good for. I therefore re-wrote and re-organized the Discussion to highlight the significance and implications of the analyses in a new section (**Place code framework in auditory processing: evidence and implications**; line 319). To summarize the contents of this section:

a. Functional units with flexible borders allows for high resolution representation of *both* frequency and sound pressure. To show this, I compare the theoretically attainable number of frequency and pressure levels with functional units that resemble classical cortical columns (lines 332-351).

b. The algebraic operations are consistent with experimental data and in some cases offer alternative interpretations. Consistent with the addition operation (defined as the union) are observations that two tones with similar frequencies are perceived as a single tone (line 369) and binaural inputs (electrophysiological and psychophysical) sum sublinearly (lines 371-380). The sublinear summation does not necessarily require inhibition. The effect of the multiplication operation (defined as the set minus) is consistent with the increase in receptive fields of neurons after inhibition is blocked (line 384). The manner in which multiplication distributes over addition suggests ways of designing experiments to examine the effects of multiple excitatory and inhibitory inputs (lines 388-405). Finally, the discretization of frequency space (via equivalence classes) is related to experimentally-measured just noticeable difference in frequency (or frequency difference limens).

c. The formal description of the neural space that supports a place code can be used to construct mappings to other brain regions along the auditory pathway (lines 406-413). It potentially sets constraints on the anatomical organization of neural spaces that will support a desired mapping.

d. The formalism is used to describe a perceptual phenomena and to infer possible network mechanisms. Algebraic operations are derived that describe loudness summation (lines 414-475), leading to a possible circuit interpretation of the so-called "critical band". Proofs in the supplementary material (lines 115-156) defines precisely the terms and sets the conditions where the formula applies. See also my response to referee 1's comment 2 for more details.

4. The author appears to make two predictions. One is that the place code is sufficient for representing sounds (line 329). It is unclear how this conclusion is derived, and what sufficient even means. Clearly, the manuscript itself states that there are limitations of the place code with regard to the resolution of sounds and ambiguity of complex sounds (lines 139-143).

I agree that this is a misleading and confusing statement. I deleted the sentence in the revised manuscript.

5. A second prediction is that the response to a complex sound is not simply a combination of responses to the simple sounds that it consists of. In other words, the manuscript states that responses are nonlinear. This appears to be a trivial consequence of using a nonlinear process of inhibition, as defined by the manuscript itself.

The sentence was poorly constructed and was not meant to refer to the simulations. The mathematical model has no biophysical variables and so the conclusion was reached based on the addition operation and the distributive properties of multiplication. The conclusion will hold generally for any model that adheres to the place coding scheme described here. I added a sentence to emphasize this point (line 403 of revised manuscript).

6. The assumption that rate coding does not work on very short timescales is questionable. Even if an individual neuron is binary (spike or no spike) within a short time interval, the firing rate can be measured in a large enough population of neurons as the probability of spiking i.e., the fraction of neurons with a certain preferred frequency that responded within a short time window.

I had considered the possibility of using a population rate coding scheme in combination with a columnar organization as an alternative means of encoding frequency and intensity (lines 335-345 of original manuscript). In this scheme, the active column provided information about the frequency and the number of active neurons within a column reflected the stimulus intensity. As the referee points out, a large enough population of neurons is needed for the population rate code to be effective. This created a problem for achieving both high resolution frequency and sound pressure representations (see also my response to comment 2 above). To increase the number of frequency levels, the columns should be as small as possible. However, this means that the number of cells in each column for intensity coding is small. This passage is now under the subsection **Representation of frequency and sound pressure** (lines 324-351) of the revised manuscript.

7. It is unclear why the manuscript defines frequency as the leftmost endpoint of an interval. Shouldn't it be the center of an interval? I.e., louder sounds should activate frequencies both to the left and to the right of a preferred frequency.

This was just for mathematical convenience as it facilitated defining of the intervals (line 401 of original manuscript). There is some evidence at least in the cochlea, that the edge rather than the center contains frequency information: an increase in the intensity causes response area to increase towards low frequency region of the basilar membrane while the high frequency cutoff remains fixed [1, 2, 3]. However, any point in the interval can be used as long as a corresponding point can be found in all the intervals that uniquely identifies each interval. The center of the interval might be more practical for experiments. Passage now in lines 495-503 of revised manuscript.

8. "unlikely" should actually be "likely" Text was changed

9. $<$ (less than) should be less than or equal to Text was changed

10. the use of subscripts is confusing... The subscripts were changed to be different in all the synaptic intervals.

References

[1] JJ Zwislocki. "What is the cochlear place code for pitch?" In: *Acta oto-laryngologica* 111.2 (1991), pp. 256–262.

[2] Monita Chatterjee and Jozef J Zwislocki. "Cochlear mechanisms of frequency and intensity coding. I. The place code for pitch". In: *Hearing research* 111.1-2 (1997), pp. 65–75.

[3] Jozef J Zwislocki and My Nguyen. "Place code for pitch: A necessary revision". In: *Acta oto-laryngologica* 119.2 (1999), pp. 140–145.
