# Supplementary material for: Mathematical framework for place coding in the auditory system
Source: PLoS Comput Biol. 2021 Aug 2;17(8):e1009251. doi: 10.1371/journal.pcbi.1009251 (PMC8360601; doi:10.1371/journal.pcbi.1009251)
Supplement: Attachment — Submitted filename: Responses to Referees_6_6.pdf [file pcbi.1009251.s003.pdf]

Responses to Referees:

Reviewer #1: I appreciate the goal of developing a mathematical approach to studying place coding in the auditory system. The author improved the manuscript with the revision, but there remain points that should be clarified.

I thank the referee for his/her careful reading the revised version of the manuscript. His/her comments forced a more thorough examination of the relation between Δf *and the difference limen* Δf_{DL}.

1a. Although my comment 3 from the first review was addressed in the manuscript, the issue continues to be a point of confusion. The author now states that experiments should be performed to test the hypothesis that the JND should range from being infinitesimally small to being equal to Δf. While it would be helpful to directly test the hypothesis, it seems unlikely that such a striking phenomenon would not have been noted in the many existing studies of frequency JND.

The main difference between Δf *and the frequency difference limen* Δf_{DL} *is that the former is fixed and is determined by the number of cells in neural space while the latter depends on several variables such as stimulus duration (Moore, JASA 1973) and intensity (Ehret, J. Comp. Physiol., 1975). Importantly, because* Δf_{DL} *is a psychological measure, it also depends on unknown sources of 'internal noise'.*

The effect of internal noise is captured by the sensitivity index d-prime (d'), which is related to the difference limen (Dai et al., 2011, JASA vol 130) in the following way. If the perceived frequencies when a tone of a given frequency is presented repeatedly is distributed normally, then:

$$d' = \frac{|\mu_{f_\alpha} - \mu_{f_\beta}|}{\sigma}.$$

where the numerator gives the difference in means of the distributions of two tones with frequencies f_α *and* f_β *and the denominator is the standard deviation (assumed identical for simplicity). The difference limen,* Δf_{DL}*, is then smallest difference in means that can be discriminated accurately (high d' value).*

The prediction that Δf_{DL} *range from be infinitesimally small to* Δf *may not have been observed in previous experiments if* Δf *is very small because* Δf_{DL} *could then span several contiguous* Δf*'s:* $\Delta f_{DL} = n\Delta f$*. The equivalent of the experiment proposed in the original manuscript is then as follows. To be detectable, two test tones with frequencies* f_α*, and* f_β*, have to be separated by* $\Delta f_{DL} = f_\beta - f_\alpha$*. If* f_α *is within the equivalence class* $[f_0, f_0 + \Delta f)$ *and* f_β *is close to the* $f_{n-1} + \Delta f$ *border (right side) of the equivalence class* $[f_{n-1}, f_{n-1} + \Delta f)$ *then a small increment* ε *in* f_β *will place it in the next equivalence class* $[f_n, f_n + \Delta f)$*. On the other hand, if* f_β *is close to the* f_{n-1} *border (left side), then* f_β *has to change by* Δf *to be placed in the next equivalence class. From the above equation, there will not be a substantial difference in discriminability (d') with* $f_\beta + \varepsilon$ *and with* $f_\beta + \Delta f$ *unless* Δf *is large and/or the internal noise* σ *is small. Consequently, phenomenon would exceedingly difficult to detect experimentally if* Δf *is small. Even if* Δf *is comparable to* Δf_{DL}*, experiments would have to be designed specifically to look for the effect and numerous trials will be needed to establish statistical significance.*

I did not to include the preceding in the revised manuscript as it is already too long. The important point is that Δf_{DL} *is a psychophysical measure and is subject to several external and internal variables whereas* Δf *is a theoretical ideal. These are concisely summarized in a short paragraph (line 423- 429) though I am willing to include the full explanation if the referee feels strongly that it is needed.*

1b. Is it possible to revise the framework so that the JND would not be sensitive to where the test frequencies fall within the intervals?

As per the discussion above, a simple fix is to let Δf *to be very small. The rationale for* Δf *was that the neural space is effectively discretized by the presence of neurons. Hence, the* Δf *is limited by* Δx*, which was initially defined to be the space taken up by a single neuron. In the schematic of Fig 2a, the interval is from the projection of a neuron in a* ***single*** *layer onto the tonotopic axis. In the supplementary text and new figure S1 of the revised manuscript (see figure below), a more general definition of* Δx *is given where the partitions of the tonotopic axis are determined by projections of staggered neurons across* ***multiple*** *layers. This scenario is also likely more realistic biologically and corresponds, for example, to the thickness of layer 4 in cortex. This does not change theory, only now* Δx*, and hence* Δf*, can be very small, depending on the density of neurons and number of layers. For very small* Δx *and* Δf*,* Δf_{DL} *will not be sensitive to the location of the test frequencies.*

I made slight changes in the wording to the main text to indicate that the Δx *has a more general definition:*

line 94: "For explanation purposes, the neural space is constructed from single row of neurons (Fig. 2a) (see Supp. text and Supp. Fig. S1 for a more general definition with multiple layers)."

line 100: "For a monolayer of neurons (Fig. 2a,b), Δx *may be viewed as the width of an individual neuron and* N_c *as the number of cells."*

line 420: "Though the model was introduced with Δx *equivalent to the diameter of a single cell in a single layer (Fig.),* Δx*, and hence* Δf*, can be much smaller if several layers of neurons are considered (Supp. Fig. S1)."*

2. The mathematical framework should make statements about coding as precise as possible. With this view, the notion that df and dp are loosely related to the JND is unsatisfying. Can the nature of this relationship be made explicit?

Δf *may be interpreted as the lower bound for* Δf_{DL}*. The text (line 120) is re-written as: "Physiologically,* Δf *and* Δp *limit the resolutions of frequency and sound pressure perception and set the lower bounds of difference limens (see Discussion)." The factors that determine* Δf *and* Δf_{DL} *are explained in the Discussion in 2 paragraphs starting at line 417. A sentence was added (line 426) that states: "For these reasons,* Δf_{DL} *is likely to be larger than* Δf*;* Δf *may be viewed as the lower bound for* Δf_{DL} *for a purely place-coding scheme that would be realized under optimal, noiseless conditions."*

3. Please clarify the definition of the neural and acoustic spaces. It appears that the spaces are defined as the collection of half-open intervals. If this is the case, then the space is not closed under the addition operation (union) because the union of two disjoint half-open intervals is not a half-open interval. It is not clear whether the spaces are the collection of half-open intervals or the collection of unions of all such intervals.

The neural space is a collection of unions of half open intervals. It is essentially the topology constructed from unions of cells (lines 39-43) of Supplementary text). Consequently, a union of any open set in the topology is again an open set. However, to make the material more easily appreciated by non-

mathematicians (as suggested by referee 4), I have removed any reference to 'closure' in the main text and direct the interested reader to the supplementary text for a more formal treatment.

The acoustic space is initially constructed from half-open intervals corresponding to frequency and pressure. After discretization, which involved partitioning into intervals of lengths Δf and Δp, only the audible frequencies and sound pressure levels (described by the sets F and P; eq. 4 of results and lines 52-56 of the supplementary text; see also Fig. 2c,d) are mapped to neural space.

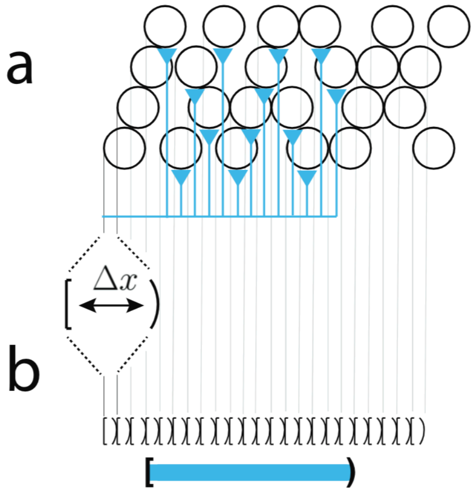

Supporting Fig. S1: Attaining subcellular spatial resolution *a*, multiple layers of staggered neurons. *b*, Projections of individual neurons partition the tonotopic axis, with Δx corresponding to the distance between the nearest neighbors on tonotopic axis. Cyan bar depicts synaptic interval generated by afferents in *a*.

Reviewer #4: This is a review of a "Mathematical framework for place coding in the auditory system" by A. Reyes. This is a resubmission, but it my first time reading this manuscript (I did not review the 1st submission).

I am very grateful to the referee for reviewing the manuscript and for considering my responses to the previous referees' comments. I have incorporated most of the referee's suggestions in the revised manuscript.

1. * this framework explicitly leaves out all temporal information of inputs and neural responses, and yet we know the auditory system is highly specialized to extract temporal information from inputs. It would be helpful for the author to expand on this in the Introduction (see some detailed comments below)

The main goal is to determine as precisely as possible what can and cannot be done with a pure place code. Consequently, all the other sources of information are omitted. As I point out in the introduction (paragraph starting line 37 of revised manuscript), much information about the sound features can already be extracted with a place code, as supported by the success of cochlear implants and there are situations where a place code is ideal (short duration sounds). I now make it clearer in the introduction and discussion that: 1) this study focuses exclusively on the place code (lines 61, 377); and 2) other schemes (temporal, rate codes) are also important for extracting detailed features of sound (line 46). I also expand the paragraph in the discussion to stress that auditory perception is significantly improved when these other cues are considered (line 477 of revised manuscript) .

2. * this framework does not account for nonlinearities that are well-known to arise for even the most basic combinations of tones (some examples: perceptual phenomena such as combination tones and missing fundamental; two-tone suppression at the the cochlear level), and also does not account for complications of phase (as raised by Reviewer 2 in the previous round). The author should discuss how the model should be interpreted in light of these phenomena

In addition to the changes in the Introduction mentioned above, I make more explicit the limitations of the model and place code in general and list specific examples of phenomena (some of which the referee points out) that cannot be accounted for by a pure place code (lines 481-488). I also stress that these variables enhance auditory perception (line 479).

3. * what is the utility (or anticipated utility) of the mathematical formalism & topological machinery introduced? It seems that that the model is defined by set operations (union, exclusion) on intervals. These are topics that could be familiar and intuitive to non-mathematically-sophisticated readers (and thus the model could be presented in a more mathematically "gentle" way, without much reference to topological terms). Of course, if the topological formulation leads to insights gained, for example, from "deeper" theorems or tools of topology, then the approach would be justified, but I don't see substantial uses of the topological approach in this manuscript. There is also mention of more esoteric algebraic structures in the supplemental material (monoid, magma) but no discussion of the significance of these structures in the current study.

I sought to use mathematical principles as a way of constraining the model and so the formalism was needed in most cases. The topology provides a mathematical architecture that essentially describes the 'functional units' of the neural space and the spatial relations between them. Here, the topology is constructed from half-open intervals of varying lengths, which represent synaptic fields generated during a stimulus (an alternative is the columnar organization with sharp bordered elements; line 396). With the

topology defined, it was possible to show that e.g. the addition operation is closed: the union of any intervals results in interval(s) that are again in the space (see response to referee 1's comment #3). The topology can also be used in future studies to define mappings across brain regions in the auditory pathway. For example, whether the mapping is continuous or bijective will depend on the topologies of the spaces. I do agree that the casual reader will not necessarily be interested in the construction and so I've deleted references to topology (except where absolutely needed) in the main text. In the supplementary text, I removed the sections referring to the topology of frequency and pressure spaces and simplified the description of the topology of neural space.

The terms monoid and magma are used simply to refer to the algebraic structures for addition and multiplication described in the text. While they are not referenced specifically in the main text, I would prefer to leave them in the supplementary text to make it easier for interested readers to do an online search; there are many sites that give simpler and more intuitive explanation of the algebraic structures.

I have also removed obscure mathematical jargon including alternate definition of multiplication as the intersection (line 179 of previous manuscript) and to closure (see below).

4. * The formal model is compared to a spiking model. It is not stated clearly what this model represents. I take it to be a model for auditory cortex (based on the author's previous work, and the mention of "auditory cortical circuits" on line 244). The author should describe the rationale for this model. In particular, could the author expand on this choice as opposed to, say, comparing the formal predictions to activation patterns of auditory nerve simulations. Several well-known auditory nerve models are freely-available (see e.g. https://github.com/mrkrd/cochlea), the elements of tonotopy and place code are already established at this stage of auditory processing, and coding of the "simple" stimuli considered here (tones, tone combinations) would likely begin in the cochlea/ANF (whereas auditory cortex may be engaged in encoding "higher-level" auditory features).

For testing the predictions of the mathematical model, the auditory cortex model developed in the Reyes lab has several advantages. First, the spiking model enables full testing of the mathematical model. While the auditory model is an excellent model in that the tonotopy is established directly from the cochlea, it does not have the excitatory and inhibitory neurons that are needed to test the algebraic operations (addition and multiplication). The cortical model parameters were derived from experiments and the connection patterns between the E and I cells are characterized fully (Levy & Reyes, J. Neurosci. 2012). Secondly, we had already performed extensive simulations showing how the excitatory and inhibitory cells interacted to generate the firing (Levy & Reyes, PLoS Comp, 2011). I was thus able to explain why the spiking model deviated from the predictions of the mathematical model such as why inhibition did not fully cancel excitation (line 277) and why addition of excitatory inputs deviated from the union operation (line 261). Third, it was easier to change the network parameters in order to test specific aspects of the model. For example, in Figs. 7 and 8, the excitatory and inhibitory inputs were artificially uncoupled so that they could be varied independently.

I give a rationale for using the cortical model in the Discussion (paragraph starting at line 538) and in two new 2 sentences in Results (line 237). I also stress the importance of using a model that incorporates a truer representation of the tonotopic axis to test the mathematical model (line 547).

*** Detailed comments ***

5. Line 8 (abstract): I think I was confused by the mention of the bijective mapping in the abstract (and

also line 128). From this statement, I expected to see stronger results about what this framework would imply for decoding. But, as is discussed (line 146, also Fig 3) there is ambiguity in decoding. I would suggest some language to clarify that the bijection is on single tones, but not combinations of tones, or something similar?

Thank you for pointing out this confusion. I removed mention of bijection from the abstract and changed the wording in the main text (line 133) to "…the mapping of ***singl****e tones onto intervals becomes bijective (one-to-one, onto)."*

6. Lines 11-12 (abstract): suggest removing "predicted", and just say "outcomes of these operations" or "resulting outcomes of these operations" [echoing some of Referee 2 and Referee 3's concerns from previous round]

I made the suggested change in wording.

7. Lines 40-45: Couldn't a counterargument be made here that acoustic information can possibly be carried by first-spike latency (Heil1997, Bizley et al 2010, etc)?

Although the Bizley study showed that first spike latency can be used to discriminate between higher and lower frequency stimuli, it would be difficult for acoustic information to be carried by first spike latency alone as there appears to be several determinants of spike latency. As the study by Heil and Irvine showed, the first spike latency varies with both the magnitude and acceleration (2nd derivative of the rising edge of the stimulus envelop; their Fig. 2B, C). If spike latency also varies with tone frequency, then there would be confusion as to what features of the sound was being encoded. Second, the mean latency does not change substantially when the level exceeded 30 dB (the curve their Fig. 2D is shallow), particularly for rapidly rising stimulus envelops that would be expected of brief stimulus. The small dynamic range would make it difficult to encode small differences in stimulus levels.

8. More generally, it seems that some discussion should be given to the fact that there are areas of the auditory system appear highly-specialized for temporal features of sounds and temporal precision in synaptic transmission, so a "place only" code (all temporal information ignored) may be an extreme way to view encoding in the auditory system.

The study was not intended to suggest that representing sound can be accomplished with only a place code. As stated above, the goal was to use the mathematical analyses to determine what can and cannot be done with a purely place code. I've included additional sentences in the Introduction to state the scope of the study (lines 46, 61) and I've expanded the Discussion (paragraph starting on line 475) to stress that other coding strategies come into play that improve coding (line 477). I also state more explicitly the disadvantages of a purely place code and what auditory phenomena it would fail to account for (lines 481-488).

9. Line 44-46: agree that "temporal or 'volley' schemes [are] difficult to implement at the level of cortex", but an alternate view is that temporal or volley information is used at some earlier stage and transformed for use by the auditory cortex, so AC does not have to implement a place code for tones. Does this matter for interpretation of the model?

The mathematical model makes specific predictions about how ensembles of neurons should behave in response to tones and combination of tones of varying frequencies and intensities. The entire analysis is predicated on these response properties so if experiments show that this is not the case, then the model

should be abandoned. For example, a combinatorial code would not necessarily evoke firing in clustered sets of neurons but could instead activate neurons that are more scattered throughout the network. This would be inconsistent with the contiguous intervals proposed in the model.

10. Line 54-56. Could add: it is also a challenge to understand how place codes can work when inputs (sounds) are dynamic and temporally complex. [but then should acknowledge that temporal dynamics are not considered in this study]

I added two sentences to incorporate the referee's suggestion:

Line 46: "However, a purely place code is likely inadequate for dynamically complex sound; indeed, coding and perception are enhanced significantly when temporal and rate cues are factored in and when longer duration stimuli are used".

Line 61: "To simplify analyses and to reveal the inherent advantages and limitations, the model focuses on how simple tones are represented and combined with a pure place code and excludes the dynamic variables that mediate temporally complex sounds."

11. General comment on Intro: would be helpful to be more explicit that the author's definition of "place code" means place ONLY, no temporal information of any kind considered in inputs or neural responses. and then comment on the meaningfulness & limitations of this approach for understanding the auditory system.

Please see responses to comments 1 and 8 above. Changes are on line 61.

12. Line 62 - "Math model" - change to mathematical

Done.

13. Eq. 3: The statements in lines 504-507 should also be included near here to clarify that f and p are not defined on a logarithmic scale, as might be expected

Done. Changes are on line 126.

14. Line 129-131 - please provide reference for statement that E and I receptive fields differ. this seems to refer to the question of "co-tuning" that has been considered by the author before, more context/explanation could be helpful here.

I was hesitant to go into too much detail as it may detract from the main concepts. The goal was just to convey that incorporating inhibition is not straightforward. Importantly, how the length of the inhibitory interval and location is determined is not crucial since they are artificially manipulated anyway to examine relations between excitatory and inhibitory intervals in the mathematical analyses and simulations. I added several references that suggest different E and I receptive fields. and can be in either co-tuned or lateral inhibitory configuration (line 134): "The mapping is complicated by the fact that there fewer inhibitory (\II) cells than excitatory (Lefort et al., 2009), may have different tuning properties (Lakunina et al., 2020), and can be in the co-tuned or lateral inhibitory configuration depending on the stimulus (Levy & Reyes, 2011)."

15. Line 136: closure is said to be "important". Not clear to me on what grounds. Important in terms of

the ensuring the model is a sensible description of neural processes? Or important because of the author's goal to adhere to formal mathematical requirements?

It was important for the formal description and was used partly to justify defining addition as the union. For example, simply adding (in the usual sense) the lengths of two intervals could generate an interval that extends beyond the neural space. I do agree that it is not really useful for non-mathematicians and so I removed reference to closure in the main text and direct readers interested in the algebraic properties to the supplementary material (lines 155, 174, 186).

16. Line 174: suggest a more specific statement: "because the set union operation has no inverse" [instead of "because there is no inverse"]

Done.

17. Fig 7: Could dashed lines for theory predictions be included here, as was done in Fig. 6, or are they too distinct?

The scales are too different. The predicted goes to (Fig. 4b, top) and starts from (bottom) zero in contrast to the simulations in Fig. 7a and b, which goes to and starts from ~25. I would prefer not to include it but I am willing to make the change if the referee feels strongly about it.

18. Line 322: typo: "applied [to] a well-known"

The sentence was removed when the figure was moved to the Results, as requested below.

19. "Relation to Difference Limens" section. This is indeed an interesting prediction based on how the model is constructed, but doesn't this prediction require the equivalence classes (partitions of acoustic and neural space) to be very "rigid"... sensitive to infinitesimally small changes that cross the boundaries of intervals. This would seem to contradict the idea (line 115) that this construction is "loose". Indeed I would agree that it more reasonable to think of the partition of acoustic and neural space as more of qualitative/approximate way of thinking about how the place code construction.

I agree that my use of "loose" is not very precise and so deleted it. As requested by referee 1, I now detail the relationship between Δf and the frequency difference limen. The difference limen Δf_{DL} measurement is affected by the unspecified sources of internal noise of subjects (please see my response to referee 1 for details). On the other hand, Δf is a theoretical ideal that is based on the number of cells in the network. As such, Δf may be interpreted as the lower bound for Δf_{DL} that can be attained under ideal, noiseless conditions. I changed the paragraph significantly to make these points. I also removed the experiment proposed in the original manuscript because it's not clear that it will be practically possible to detect (see my response to referee 1's comments 1 and 2). I am willing to include it if the referee thinks it is important.

20. Line 355 style: fDL could be f_{DL} (subscript)

Thank you for the suggestion. I've made the change.

21. Line 371: "overlapping *non-empty* intervals" [so inequality strict}

Done

22. Line 375: FRA defined in supplemental but not defined in text

It’s now defined. (line 500)

23. Line 376: typo: "equal to [the] larger of two"

Fixed

24. Line 394-397. Multiplication not right distributive. Can this be expanded on? For instance, are there certain local connectivity circuits/motifs for which this approach is more or less relevant

The ‘right distributive’ property would apply to any situation where cells receive convergent input from two types of local interneurons. This is a common scenario in cortex. I now give a specific example in auditory cortex where this occurs (line 464): two inhibitory cell types are recruited with sound stimuli and both inhibit excitatory cells (Lakunina et al., 2020).

25. Line 401-403: "response of neurons to pure tones and white noise differ". Can something more specific be said here as to how the model is "in line" with the data. The results as presented did not specifically consider the case of broadband (noise) stimuli.

The idea was that white noise would engage both excitatory and inhibitory neurons so that the response could not be predicted by looking at the responses to individual tones and then combining them later. The sentence is not important and perhaps too vague and so I deleted it.

26. Line 404: typo. "it [is] not trivially"

Fixed

27. Line 406 - 413. "Transmission of acoustic info" section could be clarified. The terms homeomorphism & homomorphism should be defined for non-specialists.
the phrase "would necessitate that the topology of the source and target brain regions be the same" is loose here... "the same" in a formal sense that there is a homeomorphism between the two? or "the same" in some way that can be interpreted in terms of the source space and neural space.
Lines 412-413 are also vague as they do not specify what constraints the author has in mind.

Keeping in line with making the text more palatable for the non-mathematicians, the paragraph was removed as it is too abstract and will detract from the main results. The more mathematical reader will readily pick up on the implication.

28. GENERAL: Nonlinearities such as missing fundamental, combination tones

These, as well as other phenomena that can’t be accounted for by a purely place coding scheme, are addressed under Assumptions and limitations, line 474-488.

29. Line 414. Algebra of loudness summations. This could be presented as a result (and moved to Results).

I’ve moved this section to the Results

30. Line 479: Could say tonotopy starts in the cochlea (von Bekesy) and auditory nerve, i.e. prior to auditory brainstem.

I’ve re-written the sentence to incorporate the referee’s suggestion. Line 490
