# Supplementary material for: Mathematical framework for place coding in the auditory system
Source: PLoS Comput Biol. 2021 Aug 2;17(8):e1009251. doi: 10.1371/journal.pcbi.1009251 (PMC8360601; doi:10.1371/journal.pcbi.1009251)
Supplement: Attachment — Submitted filename: responses_final.pdf [file pcbi.1009251.s004.pdf]

I thank the referees once again for their careful reading the manuscript. Referee 1 had no further comments and I address Referee 4's final concerns below.

Reviewer #4: The author has revised the manuscript and addressed my concerns. In particular, several of the additions (including lines 46, 61, lines 475-488) usefully define the scope and limitations of the study. The technical approach is clearly developed in the supporting documents. This material will be much appreciated by readers who want to dive deeper into the interesting and novel results presented in the paper.

1. The paper includes simulation results from a computational model. I do not see it stated if/where that code is available. Apologies if I missed it.

I have uploaded the code used for the simulation in Github: https://github.com/AlexDReyes/ReyesPlosComp.git. I added the reference to the site at line 238 and under Supporting Information at line 594.

Two comments that I expect the author can address with some further minor revisions are:

2. * lines 113-115
"Theoretically, frequencies and pressures are unbounded and can take an uncountable number of values but under physiological conditions, the audible range is likely bounded by minimum and maximum values and is finite."

A previous reviewer flagged an earlier version of this passage, noting that finite intervals can contain uncountably many elements (for real numbers, e.g.). I think this wording could still be adjusted to avoid confusion. One simple fix could be to delete "and can take an uncountable number of values"

However, my sense is that the author is purposeful in using the word "uncountable" in order to draw a contrast with the fact that the model has finitely-many elements in acoustic space. In this case, maybe say something like

Theoretically, frequencies and pressures are unbounded and can take an uncountable number of values but under physiological conditions, the audible range of tones is likely bounded by minimum and maximum values and can be partitioned (due to JNDs / difference limens) into finitely-many discriminably-different frequencies and amplitudes ...
or worded differently, to the author's liking

As suggested, I changed the sentence to "Theoretically, frequencies and pressures are unbounded and can take an uncountable number of values but under physiological conditions, the audible range is likely bounded by minimum and maximum values and consists of a finite number of discriminable frequencies and pressure levels.".

3. * First spike latency comment. Please note: I am not an expert on this particular issue of first spike latency and have no connection to the relevant work. I am following up on this point simply because I am trying to think through the correct interpretation of the model.

In the manuscript the author writes:
[lines 40-44]: This is of some significance because in this short time interval, neurons can fire only bursts of 1-2 action potentials [14, 15], indicating that neurons essentially function as binary units. Therefore, it seems likely that neither frequency nor intensity can be encoded via the firing rate of individual cells since the dynamic range would be severely limited. "

In the letter to reviewers the author wrote:
"the Bizley study showed that first spike latency can be used to discriminate between higher and lower frequency stimuli"

so perhaps it would be appropriate to to modify the phrase "it seems likely that neither frequency nor intensity can be encoded via the firing rate of individual cells" could be modified to say something like.... single spikes may carry

some information -- for instance first-spike spike latency may carry some frequency info -- but this frequency info is ambiguous (not invariant to sound pressure level) and likely restricted to narrow ranges of sound pressure level, so for simplicity we exclude this possible source of info in our construction of a place-code model

I added a sentence in the Discussion (line 395 of revised manuscript) that states "Some information may be carried by single spike latency \cites{bizley2010}; however, spike latencies depend on other variables besides frequency and does not appear to have the dynamic range to represent the full range of audible sound pressure levels \cites{heil1997}." It seems more appropriate here than in the Introduction.
